# Early Urinary Metabolomics in Patent Ductus Arteriosus Anticipates the Fate: Preliminary Data

**DOI:** 10.3389/fped.2020.613749

**Published:** 2020-12-21

**Authors:** Flaminia Bardanzellu, Cristina Piras, Alessandra Atzei, Paola Neroni, Vassilios Fanos

**Affiliations:** ^1^Neonatal Intensive Care Unit, Department of Surgical Sciences, Azienda Ospedaliero-Universitaria and University of Cagliari, Cagliari, Italy; ^2^Department of Biomedical Sciences, University of Cagliari, Cagliari, Italy

**Keywords:** metabolomics, PDA, prematurity, biomarkers, ^1^H-NMR

## Abstract

**Introduction:** In premature neonates, the persistence of hemodynamically significant ductus arteriosus (hsPDA) can be associated with short- and long-term consequences, impairing their outcome. The correct strategy of management for such condition is under debate, especially regarding contraindications and/or side effects. In recent years, metabolomics was applied to several perinatal, pediatric, and adult conditions to investigate potential biomarkers of disease, which have become useful for early diagnosis and/or therapeutic management.

**Aim of the Study:** The main purpose of our exploratory study was to asses, through ^1^H-NMR metabolomics analysis of urinary samples at birth, possible metabolic pathways differentiating, with a significant predictive power, those preterm neonates who will subsequently develop hsPDA and neonates of comparable gestational age (GA) who will undergo spontaneous ductal closure or the persistence of an irrelevant PDA (no-hsPDA). Moreover, we investigated potential prenatal or perinatal clinical factors potentially influencing the development of hsPDA.

**Materials and Methods:** We enrolled *n* = 35 preterm neonates with GA between 24 and 32 weeks; urinary samples were collected within the first 12 h of life. Patients were closely monitored regarding intensive care, respiratory support, fluid balance and administered drugs; an echocardiogram was performed at 48–72 h.

**Results:** Our results reported a significant correlation between lower GA at birth and the development of hsPDA. Moreover, neonates with GA ≤ 30w developing hsPDA were characterized by lower Apgar scores at 1′ and 5′, higher rates of perinatal asphyxia, higher need of delivery room resuscitation and subsequent surfactant administration. Interestingly, metabolomics analysis at birth detected a clear separation between the ^1^H-NMR urinary spectra of subjects GA ≤ 30w not developing hsPDA (*n* = 19) and those of subjects born at GA ≤ 30w in which hsPDA was confirmed at 48–72 h of life (*n* = 5).

**Conclusions:** This is the first study applying metabolomics to investigate the PDA condition. Although preliminary and conducted on a limited sample, our results reveal that metabolomics could be a promising tool in the early identification of hsPDA, potentially superior to the clinical or laboratory predictive tools explored to date and even to the clinical observations and correlations in our sample, through the detection of specific urinary metabolites.

## Introduction

Ductus arteriosus (DA) or ductus Botalli, one of the fetal shunts allowing oxygenation during intrauterine life (1, 2), physiologically undergoes spontaneous functional closure within the first 72 h of postnatal life, through molecular mechanisms that have been extensively discussed (3–5).

In preterm neonates, ductal closure can fail or delay, mostly due to reduced response to oxygen-induced vasoconstriction, lower activity of vasavasorum, higher levels of prostaglandin E2 (PGE2) and increased sensitivity to prostaglandins and nitric oxide (NO) compared to full term neonates (2, 3). A low-grade inflammatory status and oxidative stress could also take part in maintaining PDA, *via* increasing prostaglandin levels (1, 6).

The risk of developing a persistence of ductal patency (PDA) increases with decreasing gestational age (GA), with relevant short- and long-term consequences in term of morbidity, especially in case of hemodynamically significant PDA (hsPDA) (1, 7, 8).

PDA closure can be performed pharmacologically or, in case of failure or contraindication, with surgical ligation or transcatheter closure (8–18).

However, all these strategies can have side effects and potential risks and, as evidenced by current literature, the ideal strategy of management is still under debate, especially regarding if, when and how to treat or not PDA.

Several predisposing factors have been associated to PDA condition, sometimes with contrasting results, such as genetic factors (19–21), maternal pregnancy induced hypertension (PIH) (22), chorioamnionitis (23, 24), maternal antepartum hemorrhage, respiratory distress syndrome (RDS), lower birth weight (BW), Apgar score and GA, female gender (22, 25), dopamine administration and eccessive intravenous fluid administration (26), neonatal sepsis (27, 28), need for surfactant (22, 25, 26, 29), maternal and neonatal drug assumption (2, 30–38), platelet number, function and other platelet-related parameters (4, 5, 39–47).

Objects of study have also been the factors that can influence therapy response, as reported for male gender (48), polymorphisms influencing drug metabolism (19, 49), and GA (48).

The full comprehension of all the molecular and genetic mechanisms underlying ductal closure or patency maintenance could help in identifying serum or urinary biomarkers potentially associated with PDA condition, especially hsPDA, supporting or anticipating ultrasound or clinical diagnosis.

HsPDA determines ductal diastolic steal, leading to systemic hypoperfusion (cerebral, renal, pulmonary, gastro-intestinal etc…) and the subsequent hypoxia can affect tissues and endothelium with variable impact on hemodynamics. Identifying early markers of this condition could help in the precocious identification of those newborns at high risk of hsPDA, orienting clinical strategy, and avoiding overtreatment of subjects who would undergo spontaneous ductal closure, improving diagnosis and treatment.

Up to now, several literature studies have tried to associate different biomarkers to hsPDA, such as serum B-type Natriuretic Peptide (BNP), segment of the amino terminal B-type Natriuretic Peptide (NT-proBNP) (1, 50–59), cardiac Troponin T (cTnT) (57), lactate levels (60), ions (61), osmolality (62), base excess or hematocrit (63), platelets count and functions (64–66), perfusion index (67), proteomics (68), and among urinary markers, isoprostans, u-ngal (69), proBNP and NT-proBNP-to-creatinine ratio (70–73), also with potential associations with PDA diameter and therapy response, often with contrasting results (74, 75).

Systemic inflammation (76–78) and oxidative stress (79, 80) have also been associated with PDA and therapy response, through urinary and serum markers.

Finding biomarkers that are able to predict therapy response could be useful to avoid unnecessary treatment and potentially perform a targeted therapy, choosing and treating only those infants that could respond to the treatment.

Metabolomics is one of the “omics” technologies that, in the last years, emerged as a promising tool to be applied in several fields of neonatology and pediatrics to characterize and describe pathophysiological processes (81, 82).

Due to its ability in dynamically detecting, in a non-invasive way, the whole set of low molecular weight molecules (including sugar, aminoacids, lipids etc…) in cells, tissues and biological fluids, it can help in describing, in a highly individualized way, the exact response of an organism to environmental stimuli and pathophysiological conditions, including drugs, nutrition, lifestyle, diseases and others, as recently reviewed by our group (81).

Metabolomics biomarkers, if accurately identified and confirmed through repeated studies and validation tests, could be investigated to obtain, in a precocious and non-invasive way, information regarding disease onset, progression and, potentially, therapy response.

Our research group has been interested in metabolomics for many years, carrying many promising studies on several prenatal, perinatal, infant, and adult conditions and diseases, with the aim of achieving a personalized medicine. More details can be found in our reviews on the topic (81, 82).

Even if, to date, the exact meaning of each metabolite variation and its pathophysiological implication was not completely defined, we strongly believe that the clinical interpretation of the promising results obtained could be completed by creating a specific and updated atlas.

Due to these reasons, we hypothesize that metabolomics could provide useful biomarkers even in the condition of PDA. Thus, we decided to perform an exploratory study analyzing urinary samples at birth in a cohort of preterm neonates, to assess, among the complete overview of possible prenatal or perinatal factors taken into account, if some metabolites could result as predictive of hsPDA, highlighting substantial differences among those neonates who subsequently will develop hsPDA and those who will undergo spontaneous closure, or in which PDA will not determine hemodynamic consequences.

To the best of our knowledge, this is the first metabolomics study in the investigation of PDA disease. A previous preliminary report by our group was performed few years ago (83) and a preliminary investigation on metabolomics differences between urinary profiles of responders and non-responders to ibuprofen therapy was performed by Castell-Miñana et al. (84).

## Aim of the Study

Our hypothesis was to identify, through potential early metabolic perturbations in the group of newborns that will develop hsPDA, different urinary pathways compared to those newborns in which the duct will undergo spontaneous closure or minimal patency without impact on hemodynamics.

Thus, the principal aim of the exploratory study was to investigate, for the first time, the possible predictive role of ^1^H-NMR metabolomics analysis of urinary samples at birth regarding the development of hsPDA. In fact, we investigated possible metabolic differences between urinary pathways of those preterm neonates who will subsequently develop hsPDA confirmed at 48–72 h of life, rather than neonates of comparable GA who will undergo spontaneous closure or the persistence of irrelevant PDA at the same time point (no-hsPDA).

We also aimed to evaluate whether the predictive role of urinary metabolomics may be superior to the clinical criteria or biomarkers considered up to now. Therefore, we investigated potential prenatal or perinatal clinical factors potentially influencing the development of hsPDA in our sample group.

Finally, we tried to find a potential link correlating metabolic variation in the two groups and pathogenesis of hsPDA; in our opinion, the early identification of specific metabolites suggestive of PDA could be a fundamental and complementary tool to be used, alongside the clinic, for applying a personalized approach to the treatment of PDA.

## Materials and Methods

### Study Population

A population of *n* = 35 preterm neonates admitted to Neonatal Intensive Care Unit of Duilio Casula Hospital, University of Cagliari, with GA included between 24 and 32 weeks, was enrolled between October 2019 and June 2020.

In all the newborns, a urinary sample of 3 mL was collected until 12 h after birth, through the non-invasive technique of the cotton wad.

All the subjects underwent cardiologic evaluation performed by the neonatal cardiologist of our neonatal intensive care unit (NICU) within the first 24 h and successively at 48–72 h and 120 h after birth. Echocardiography and Color-doppler aimed to evaluate cardiac anatomy and functionality and to assess the presence and the hemodynamic features of PDA. In subjects with a persistent PDA or hsPDA, an echocardiography was performed in the subsequent days, according to the clinical indication.

Each echocardiography aimed to asses PDA diameter (indexed for body weight expressed in kilograms), left atrium/Aortic Root Ratio (LA/Ao), patent ductus arteriosus/left branch of pulmonary artery ratio (PDA/LPA), systolic and diastolic velocity of duct flow to assess the restrictive or non-restrictive pattern. Additional cardiological evaluations were performed according to clinical necessities. PDA was considered hemodynamically significant in case of a PDA diameter/neonatal weight ≥ 1.5 mm/Kg and LA/Ao > 1.4 in addition to an eventual PDA/LPA > 0.8 and/or a peak diastolic velocity lower than 50% of the peak systolic velocity measured at ductal level 48–72 h after birth.

Echocardiography was performed using Esaote MyLab™Twice EVO 13.0/13.0 m, and the images' acquisition was obtained with 8 MHz two-dimensional probe.

During hospitalization, all the newborns underwent instrumental exams (chest radiography, cerebral ultrasound, abdominal ultrasound, hearing screening, fundus oculi exam) and hematic exams (complete blood count, hepatic and renal function, infection indexes, and blood culture in case of suspected sepsis), eventually repeated according to the clinical needs.

All demographic data and the information regarding pregnancy, birth, neonatal management in delivery room, assistance in NICU, invasive and non-invasive ventilation, oxygen needs, surfactant administration, fluid balance, nutrition, and drugs were recorded.

The Ethics Committee of University of Cagliari gave its approval for this study (Protocol NP/2017/470) and the parents signed an informed consent and gave their approval for this study.

### Sample Analysis

#### Urine Sample Preparation

Urine samples were thawed in ice. An aliquot of 800 μL of urine was transferred into a tube together with 8 μL of a 1% aqueous solution of NaN3 in order to inhibit bacteria growth and stored at −80°C. The sample was centrifuged at 12,000 g for 10 min at 4°C to remove solid particles. Then, 630 μL of the supernatant solution was mixed with 70 μL of potassium phosphate buffer in D2O (1.5 M, pH 7.4) containing sodium 3-trimethylsilyl-propionate-2,2,3,3,-d4 (TSP) as an internal standard (98 atom% D, Sigma-Aldrich, Milan). The mixture was vortexed and an aliquot of 650 μL was transferred to 5-mm NMR glass tubes for ^1^H-NMR analysis (85).

#### ^1^H-NMR Spectroscopic Analysis and Data Pre-processing

^1^H-NMR spectra were recorded at 300 K using a Varian UNITY INOVA 500 spectrometer operating at 499.839 MHz for proton and equipped with a 5 mm double resonance probe (Agilent Technologies, CA, USA). The acquisition parameters of the ^1^H-NMR spectra are reported in our previous article (85). NMR spectra were processed using an ACDlab Processor Academic Edition (Advanced Chemistry Development, 12.01, 2010). After Fourier transformation with 0.3 Hz line broadening, ^1^H-NMR spectra were phased and baseline corrected and chemical shifts referenced to the signal of internal standard TSP at δ= 0.0 ppm. The spectral region comprising the signal of residual water and urea (4.7–4.9 ppm) was removed. The ACD Labs intelligent bucketing method was used for spectral integration (86). A 0.04 ppm bucket has been applied. The area of bucket regions were normalized to the total spectral area to compensate the different dilutions of original urine samples. Finally, the spectral data was imported into the SIMCA software (Version 15.0, Sartorius Stedim Biotech, Umea, Sweden) for statistical multivariate analysis. All imported data were then pre-processed using Pareto scaling (87).

### Statistical Analysis

The data were collected and analyzed with Microsoft Excel 2019 program; the results were expressed as the mean value ± the standard deviation (SD). For statistical analysis, Student's *T*-test was applied to compare the means of each variable analyzed in the study. A *p*-value < 0.05 was considered as statistically significant, and a *p*-value < 0.001 as highly significant.

A Chi-Square-test was used for testing relationships on categorical variables and to evaluate tests of independence.

#### Multivariate Statistical Analysis

Principal component analysis (PCA), Partial Least-Squares Discriminant Analysis (PLS-DA) and Orthogonal Partial Least-Squares Discriminant Analysis (OPLS-DA) were used for multivariate statistical analyses of NMR data (88). Principal component analysis is an unsupervised analysis and is applied to identify unusual clusters, anomalies, or trends in the samples based on the similarities of their metabolic profiles. Supervised PLS-DA and OPLS-DA methods were used to reduce model complexity and to better highlight variables responsible for the discrimination of predefined classes. The models quality expressed as R2Y (goodness of classification) and Q2Y (goodness of prediction) and the over-fitting were evaluated through the default leave-1/7th-out cross validation test and “permutation test” (400 times) respectively. The intercept is a measure of the overfit, Q2Y intercept value <0.05 is indicative of a valid model. To identify potential metabolites that mainly contributed to group separation, an S-line plot for the OPLS-DA models was built (89).

#### Univariate Statistical Analysis

GraphPad Prism software (version 7.01, GraphPad Software, Inc., San Diego, CA, USA) was used to perform the univariate statistical analysis. The potential significant metabolites identified were quantified using Chenomx NMR Suite 7.1 (Chenomx Inc., Edmonton, Alberta, Canada) (90). The statistical significance of the differences in metabolite concentrations was calculated by using the Mann-Whitney U-test and a *p*-value < 0.05 was considered statistically significant. The Benjamini-Hochberg method was subsequently applied to the obtained *p*-values to acquire the level of significance for multiple testing (91). Finally, to further evaluate the diagnostic power of the potential biomarkers the ROC (receiver operator characteristic) curve was constructed to test the sensitivity, specificity and calculate area under the ROC curve (AUC) to only metabolites with *p*-values < 0.01. AUC indicates the accuracy of a test for correctly distinguishing No-hsPDA from hsPDA. The 95% CI (Confidence Interval) for the AUC curves were also calculated.

## Results

### Clinical Analysis

Our study population was composed by *n* = 35 newborns with GA included between 24^+2^ and 32^+4^ weeks. Mean GA was 29.92 ± 2.06 weeks. Mean birth weight was 1,346 ± 367 g and *n* = 23/35 subjects were males.

All the information regarding pregnancy characteristics, complications and outcome are reported in Table 1.

**Table 1:** Maternal and pregnancy characteristics and complications.

		**Total sample** **(≤32 w)**	**≤30 w**	**≤30 w** **no-hsPSA**	**≤30 w** ** hsPDA**	*p-*value^*^ (calculated comparing no-hsPDA and hsPDA)
Chorioamnionitis	Yes	2/35 (5.71%)	2/24 (8.33%)	2/19 (10.53%)	0/5 (0%)	0.3
	No	33/35 (94.29%)	22/24 (91.67%)	17/19 (89.47%)	5/5 (100%)	
Altered glucose load curve	Yes	1/35 (2.86%)	1/24 (4.17%)	1/19 (5.26%)	0/5 (0%)	-
	No	16/35 (45.71%)	8/24 (33.33%)	6/19 (31.58%)	2/5 (40%)	
	N.p.	18/35 (51.43)	15/24 (62.5%)	12/19 (63.16%)	3/5 (60%)	
Placental abruption	Yes	6/35 (17.14%)	6/24 (25%)	4/19 (21%)	2/5 (40%)	0.4
	No	29/35 (82.86%)	18/24 (75%)	15/19 (79%)	3/5 (60%)	
Hypertension (in treatment)	Yes	4/35 (11.43%)	4/24 (16.67%)	2/19 (10.53%)	2/5 (40%)	0.11
	No	31/35 (88.57%)	20/24 (83.33%)	17/19 (89.47%)	3/5 (60%)	
PROM > 18 hours	Yes	5/35 (14.29)	5/24 (20.3%)	5/19 (26.31%)	0/5 (0%)	0.19
	No	30/35 (85.71%)	19/24 (79.17%)	14/19 (73.69%)	5/5 (100%)	
Antenatal Steroids	Yes	24/35 (68.57%) 2 doses: 22/24 1 dose: 2/24	15/24 (62.5%) 2 doses: 14/15 1 dose: 1/15	12/19 (63.16%) 2 doses: 11/12 1 doses: 1/12	3/5 (60%) 2 doses: 3/5	0.9
	No	11/35 (31.43%)	9/24 (37.5)	7/19 (36.84%)	2/5 (40%)	

*w, Weeks; hsPDA, hemodinamically significant patent ductus arteriosus; n.p., not performed; PROM, premature rupture of membranes (>18 h)*.

* *Statistically significant if p < 0.05*.

- *, Not calculated because the authors do not believe it could be useful for statistical analysis*.

In our sample of preterm neonates, *n* = 4/35 were born by vaginal delivery (VD) and *n* = 31/35 by urgent cesarean section (UCS). On a total of *n* = 29 pregnancies, *n* = 3 deliveries were vaginal, while *n* = 26 were UCS. The causes of preterm birth through UCS have been represented by intrauterine growth restriction (IUGR) and flux alteration (23%), cardiotocography (CTG) alterations/neonatal bradycardia (23%), placental abruption (19.2%), preterm labor/other maternal causes (19.2%), preeclampsia (8%), twin-to-twin transfusion (3.8%), and fetal tachycardia (3.8%).

All the information regarding perinatal characteristics of our group of newborns have been reported in Table 2 and neonatal outcomes, as well as the information regarding the development of hsPDA and the needs for treatment are reported in Table 3. In *n* = 5/35 neonates (14.3%) hsPDA was confirmed at echocardiographic examination at 48–72 h of life. All the newborns developing hsPDA were born at GA ≤ 30weeks. Thus, to make the sample more homogeneous, we decided to report our results excluding those neonates of GA > 30 weeks, since hsPDA resulted as being highly dependent on GA at birth and was absent in neonates > 30 weeks. However, all the results obtained by the analysis of the whole population can be found in Tables 1–3.

**Table 2:** Perinatal characteristics.

		**Total sample** **(≤32 w)**	**≤30 w**	**≤30 w** **no-hsPSA**	**≤30 w hsPDA**	*p-*value^*^ (calculated comparing no-hsPDA and hsPDA)
	Number of subjects	35	24	19/24 (79.2%)	5/24 (20.8%)	<0.01^*^
	Sex	M 23/35 (65.7%)	M 14/24 (58.33%)	M 11/19 (57.9%)	M 3/5 (60%)	0.93
	Gestational age	w ± SD 29.92 ± 2.06	29 ± 1.85	29.63 ± 1.03	26.62 ± 2.46	<0.001^*^
	Birth weight g ± SD	1,346 ± 367	1239.92 ± 329	1,302 ± 261	1,004 ± 476	0.069
Twins	Yes	13/35 (37.14%)	9/24^**^ (37.5%)	9/19 (47.4%)	0/5 (0%)	<0.05^*^
	No	22/35 (62.86%)	15/24 (62.5%)	10/19 (52.6%)	5/5 (100%)	
Antropometry (percentiles)	LGA	4/35 (11.4%)	3/24 (12.5%)	2/19 (10.5%)	1/5 (20%)	0.75
	AGA	29/35 (82.9%)	20/24 (83.3%)	16/19 (84.2%)	4/5 (80%)	
	SGA	2/35 (5.7%)	1/24 (4.2%)	1/19 (5.3%)	0/5 (0%)	
Mode of delivery	VD	4/35 (11.43%)	1/24 (4.2%)	1/19 (5.26%)	0/5 (0%)	0.6
	UCS	31/35 (88.57%)	23/24 (95.8%)	18/19 (94.74%)	5/5 (100%)	
Apgar Score Mean ± SD	1′	5.83 ± 2.25	6 ± 2.32	6.58 ± 1.74	3.8 ± 3.11	<0.01^*^
	5′	7.34 ± 1.97	7.46 ± 1.67	7.48 ± 1.21	6 ± 2.45	<0.05^*^
Apgar Score (Median)	1′	6	6	7	3	**-**
	5′	8	8	8	5	**-**
Delivery room resuscitation	No	2/35 (5,7%)	1/24 (4,17)	1/19 (5,26%)	0/5 (0%)	<0.05^*^ e.t intubation: <0.05^*^
	n.i. ventilation + O_2_	25/35 (71.4%)	18/24 (75%)	16/19 (84.21%)	2/5 (40%)	
	e.t. intubation + O_2_ ± chest compressions or adrenaline	8/35 (22.9%)	5/24 (20.83%)	2/19 (10.53%)	3/5 (60%)	
Perinatal asphyxia	No	18/35 (51.4%)	12/24 (50%)	10/19 (52.64%)	1/5 (20%)	<0.05^*^ Severe: <0.01^*^
	Mild	12/35 (34.3%)	9/24 (37.5%)	8/19 (42.1%)	1/5 (20%)	
	Severe	5/35 (14.3%)	3/24 (62.5%)	1/19 (5.26%)	3/5 (60%)	

*w, Weeks; hsPDA, hemodinamically significant patent ductus arteriosus; g, grams; SD, standard deviation; LGA, lrge for gestational age; AGA, appropriate for gestational age; SGA, small for gestational age; VG, vaginal delivery; UCS, urgent cesarean section; n.i., non-invasive; e.t., endotracheal*.

* *Statistically significant if p < 0.05*.

** *Twins are not even in number, since one of them was excluded by the study because we could not collect the urinary sample. Bold values are statistically significant*.

**Table 3:** Neonatal early outcome.

		**Total sample** **(≤32 w)**	**≤30 w**	**≤30 w no-hsPSA**	**≤30 w hsPDA**	*p-*value^*^ (calculated comparing no-hsPDA and hsPDA)
RDS		35/35 (100%)	24/24 (100%)	19/19 (100%)	5/5 (100%)	-
Need for surfactant	Yes	22/35 (62.9%)	15/24 (62.5%)	10/19 (52.63%)	5/5 (100%)	<0.05^*^
	No	13/35% (37.1%)	9/24 (37.5%)	9/19 (47.37%)	0/5 (0%)	
Surfactant doses (*n*)	1	14/35	8/24	7/19	1/5	-
	2	7/35	5/24	3/19	2/5	-
	4	1/35	1/24	0/19	1/5	-
hsPSA	Yes	5/35 (14.3%)	5/24 (20.83%)	0/19 (0%)	5/5 (100%)	-
	No	30/35 (85.7%)	19/24 (79.17%)	19/19 (100%)	0/5 (0%)	-
Treatment for hsPDA	Yes	4/35 (11.4%)	4/24 (16.67%)	0/19 (0%)	4/5 (80%)	-
	No	31/35 (88.6%)	20/24 (83.33%)	19/19 (100%)	1/5 (20%)	-
hsPDA closure^**^	Yes (treatment)	4/35 (11.42)	4/24 (16.67%)	-	4/5 (80%)	-
	Yes (no treatment)	1/35 (2.8%)	1/24 (4.17%)	-	1/5 (20%)	-
	No (treatment)	0/35 (0%)	0/24 (0%)	-	0/5 (0%)	-
Sepsis	EOS	7/35 (20%)	4/24 (16.67%)	2/19 (10.53%)	2/5 (40%)	0.11
	LOS	6/35 (17.1%)	6/24 (25%)	5/19 (26.31%)	1/5 (20%)	0.77
	EOS + LOS	4/35 (11.43%)	4/24 (16.67%)	2/19 (10.53%)	2/5 (40%)	0.11
Early aminoglycosides		8/35	7/24	5/19	2/5	0.5
administration		(22.8%)	(29.17%)	(26.31%)	(40%)	
Creatinine values at 3-5 days of life mean value ± SD		0.56 ± 0.2	0.59 ± 0.21	0.57 ± 0.21	0.65 ± 0.21	0.46
Death		0/35 (0%)	0/24 (0%)	0/19 (0%)	0/5 (0%)	-

*w, Weeks; hsPDA, hemodinamically significant patent ductus arteriosus; RDS, respiratory distress syndrome; n, number; EOS, early onset sepsis; LOS, late onset sepsis; SD, standard deviation*.

* *Statistically significant if p < 0.05*.

** *When pharmacological treatment for hsPDA was performed, ibuprofen at the standard dose was used as first choice drug, while in case of contraindication, paracetamol was preferred*.

- *, Not calculated because the authors do not believe it could be useful for statistical analysis. Bold values are statistically significant*.

Our group on neonates born at GA ≤ 30weeks was composed by *n* = 24 neonates. All the information on pregnancy, perinatal characteristics and neonatal early outcome regarding this subpopulation of newborns were analyzed and statistical comparisons were made between those newborns with GA ≤ 30weeks subsequently undergoing spontaneous closure of PDA or maintaining an hemodynamically irrelevant PDA (no-hsPDA) and those neonates developing hsPDA (Tables 1–3).

The first group, (≤30 w no-hsPDA) was composed by *n* = 19/24 neonates, while the second group (≤30 w hsPDA) included *n* = 5/24 neonates, *p* < 0.01.

Maternal/pregnancy complications were analyzed, including chorioamnionitis, placental abruption, hypertension in treatment, premature rupture of membranes (PROM > 18 h), and not statistically significant differences occurred in the two groups ≤30 w no-hsPDA vs. ≤30 w hsPDA (Table 1).

Maternal administration of steroids occurred in *n* = 15/24 mothers, *n* = 12/19 in the group ≤30 w no-hsPDA vs. *n* = 3/5 in the group ≤30 w hsPDA, *p* = 0.9.

Information regarding maternal glucose load was available only for a few patients, since such a test is performed between 26 and 28 GA and many of the women delivering preterm were not able to perform it. Thus, statistical evaluations were not performed regarding eventual alteration in glucose tolerance or pregnancy induced diabetes mellitus.

In the group of neonates ≤30 w, mean GA was 29 ± 1.85 weeks and mean BW was 1239.92 ± 329 g; *n* = 14/24 were males.

Twins were *n* = 9/24; twins are not even in number, since one of them was excluded by the study because we could not collect the urinary sample.

GA was significantly higher in the ≤30 w no-hsPDA group (29.63 ± 1.03) vs. ≤30 w hsPDA (26.62 ± 2.46), with statisticalsignificance *p* < 0.001 and even BW was higher in the fist group 1,302 ± 261 g vs. 1,004 ± 476 g, without statistical significance (*p* = 0.069).

Twins were more frequent in the group ≤30 w no-hsPDA *n* = 9/19 vs. ≤30 w hsPDA *n* = 0/5, p < 0.05.

Information regarding length, weight, and head circumference percentiles at birth were not statistically significant in terms of number of patients large for GA (LGA), appropriate (AGA) or small for GA (SGA) (*p* = 0.75). Moreover, even delivery mode in terms of VD or UCS was not statistically different in the two groups (*p* = 0.6).

Significant differences occurred, taking into account the Apgar score in the two groups of patients. Apgar score was significantly higher in the ≤30 w no-hsPDA group (6.58 ± 1.74) vs. ≤30 w hsPDA (3.8 ± 3.11), at the first minute of life (1′), with statistical significance *p* < 0.01 and at 5 min of life (5′) (7.48 ± 1.21) vs. (6 ± 2.45), *p* < 0.05. The incidence of perinatal asphyxia was higher in the ≤30 w hsPDA group than ≤30 w no-hsPDA group (*p* < 0.05); in detail, severe asphyxia was more frequent in the first group (*p* < 0.01).

The needs for delivery room resuscitation was higher in the ≤30 w hsPDA group (*p* < 0.05), especially regarding the needs of endotracheal intubation, chest compressions, or adrenaline, occurring in *n* = 2/19 (10.53%) in the ≤30 w no-hsPDA group, vs. 3/5 (60%) in the ≤30 w hsPDA group (*p* < 0.05).

RDS occurred in the whole sample of newborns ≤30 w *n* = 24/24 (100%).

The need for one or more doses of surfactant was *n* = 10/19 in the ≤30 w no-hsPDA group and *n* = 5/5 in the ≤30 w hsPDA group (*p* < 0.05).

In the group ≤30 w hsPDA group, *n* = 4/5 (80%) were pharmacologically treated for hsPDA closure.

HsPDA closure was achieved in the whole ≤30 w hsPDA group. In *n* = 4 (20%) it occurred with treatment, while *n* = 1/5 underwent spontaneous closure (20%).

In our sample, early onset sepsis (EOS) was detected through blood culture in *n* = 2/19 among the ≤30 w no-hsPDA neonates vs. *n* = 2/5 among the ≤30 w hsPDA (*p* = 0.11); late onset sepsis (LOS) occurred in *n* = 5/19 vs. *n* = 1/5 (*p* = 0.77) and the co-occurrence of EOS + LOS in 2/19 and 2/5 (*p* = 0.11) in the same groups.

Early aminoglicosydes administration to treat EOS was administered in *n* = 5/19 among neonates ≤30 w no-hsPDA vs. *n* = 2/5 in the ≤30 w hsPDA group (*p* = 0.5), not statistically significant. Serum creatinine values at 3–5 days of life were 0.57 ± 0.21 mg/dl in the ≤30 w no-hsPDA vs. 0.65 ± 0.21 mg/dl in the ≤30 w hsPDA group (*p* = 0.46).

None of our patients died.

### Metabolomics Analysis

The metabolomics profile of urine samples was investigated using ^1^H-NMR spectroscopy coupled with multivariate data analysis. Metabolites were identified on the basis of literature information (85) and by using a dedicated library, such as the Human Metabolome Database (HMDB, http://www.hmdb.ca) and the 500 MHz library from Chenomx NMR suite 7.1. Major NMR resonances were originated from free amino acids, organic acids, sugars, small organic compounds and osmolytes. A first unsupervised PCA analysis was conducted to identify potential outliers (outside the 95% confidence limit). The result of the PCA analysis (data not shown) shows a rather homogeneous cohort of samples and no NMR spectrum was excluded from subsequent analyses. To remove potential information not related to the pathology of interest and highlight possible differences in metabolomics urine profile between No-hsPDA (≤ and >30 w) and hsPDA (≤30 w) subjects, a PLS-DA analysis was subsequently conducted on the same dataset.

**Figure 1:**
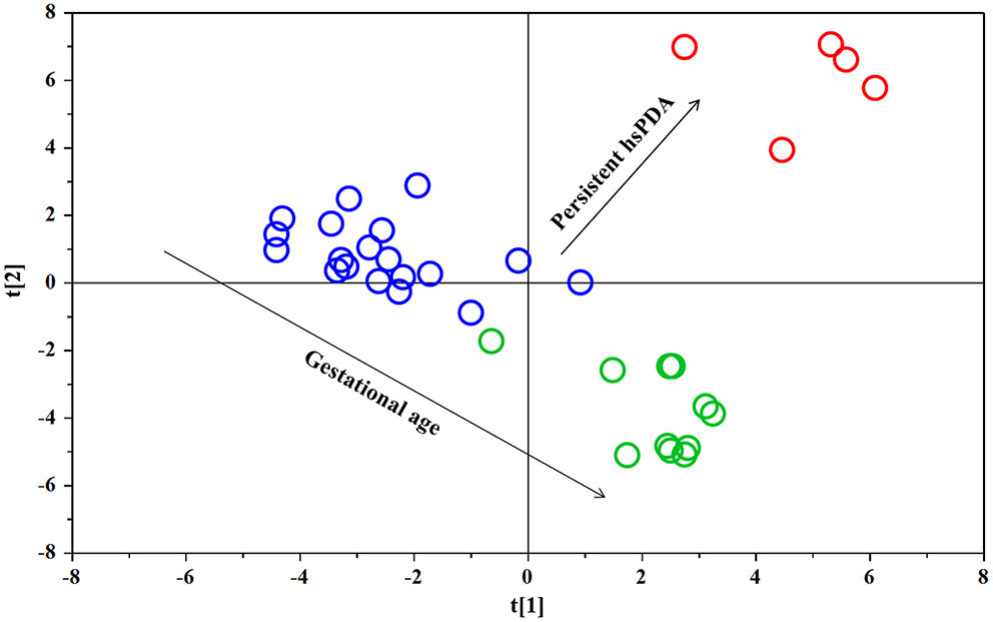
PLS-DA scores plot of ^1^H NMR spectra of urine samples: ≤30 w No-hsPDA (blue circle), >30 w No-hsPDA (green circle) and ≤30 w hsPDA (red circle).

**Figure 2:**
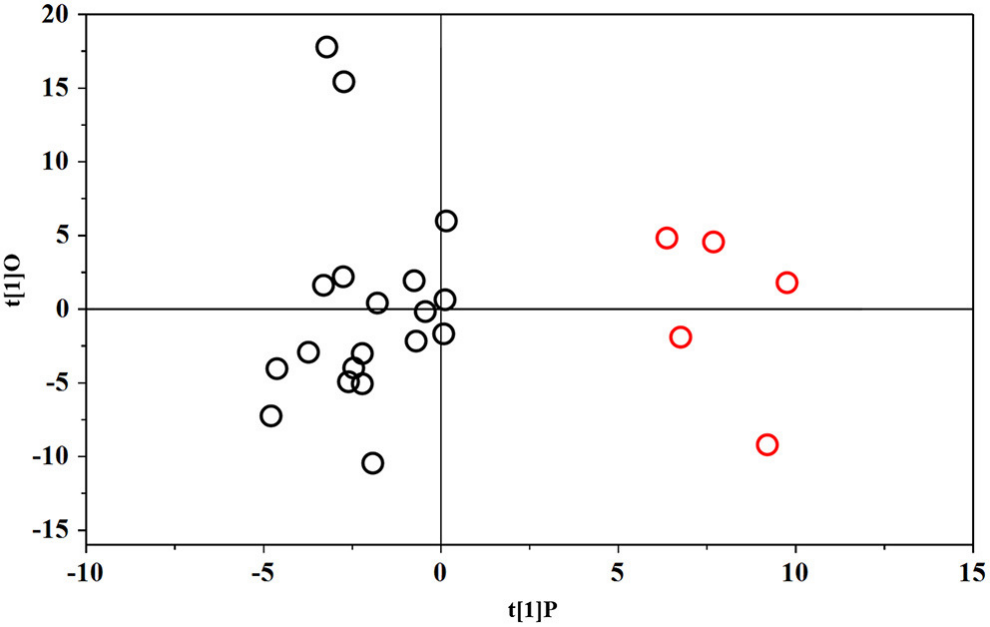
OPLS-DA scores plot of ^1^H NMR spectra of urine samples: ≤30 w No-hsPDA (black circle) and ≤30 w hsPDA (red circle).

**Figure 3:**
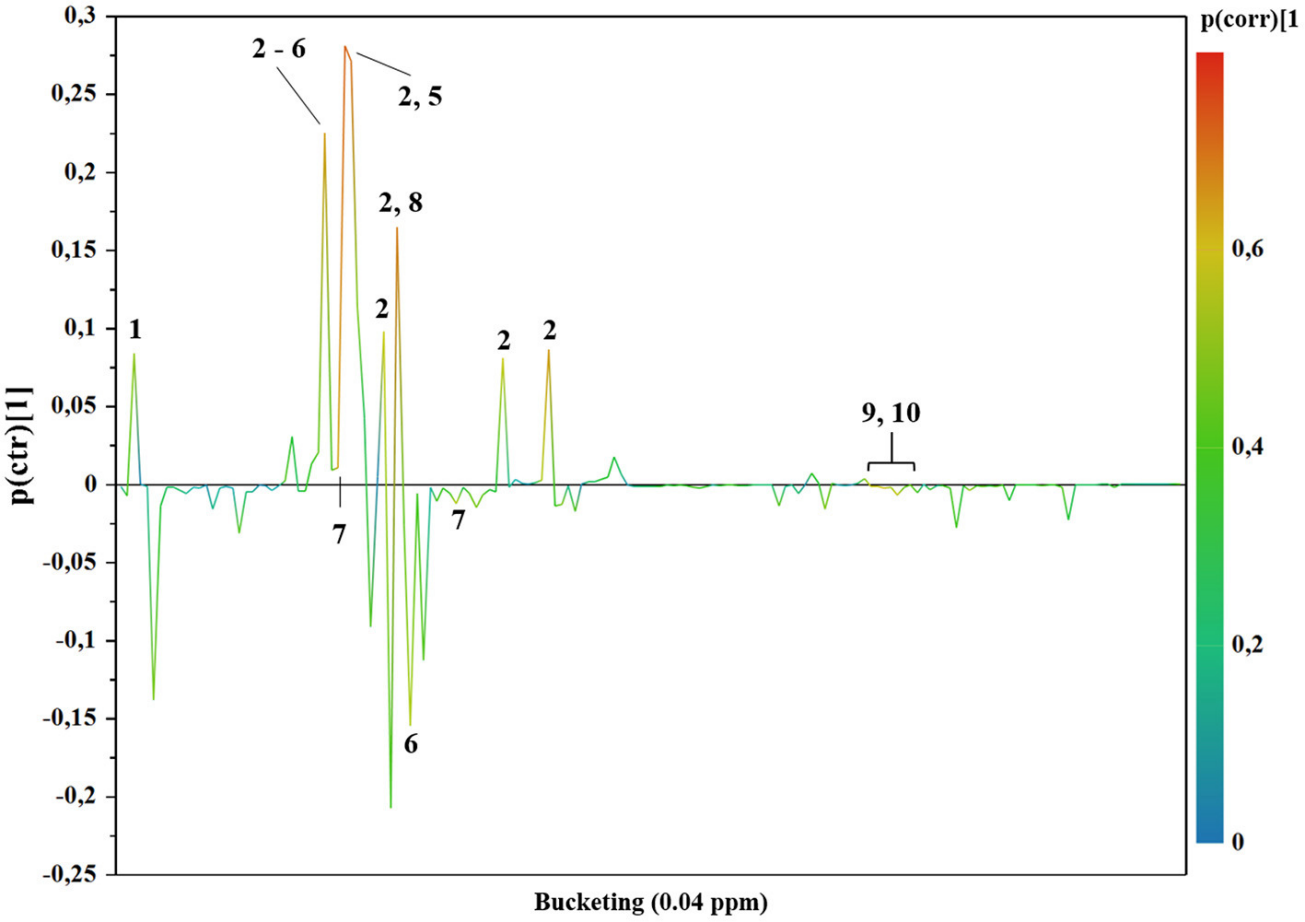
Color-coded coefficient loadings plots of urine metabolomics profiles between ≤30 w No-hsPDA and ≤30 w hsPDA subjects. Peaks: 1, Lactate; 2, Glucose; 3, Betaine; 4, Trimethylamine-N-Oxide (TMAO); 5, Taurine; 6, Myo-Inositol; 7, 4-Hydroxyproline; 8, Creatine; 9, Tryptophan and 10 Hippurate.

**Figure 4:**
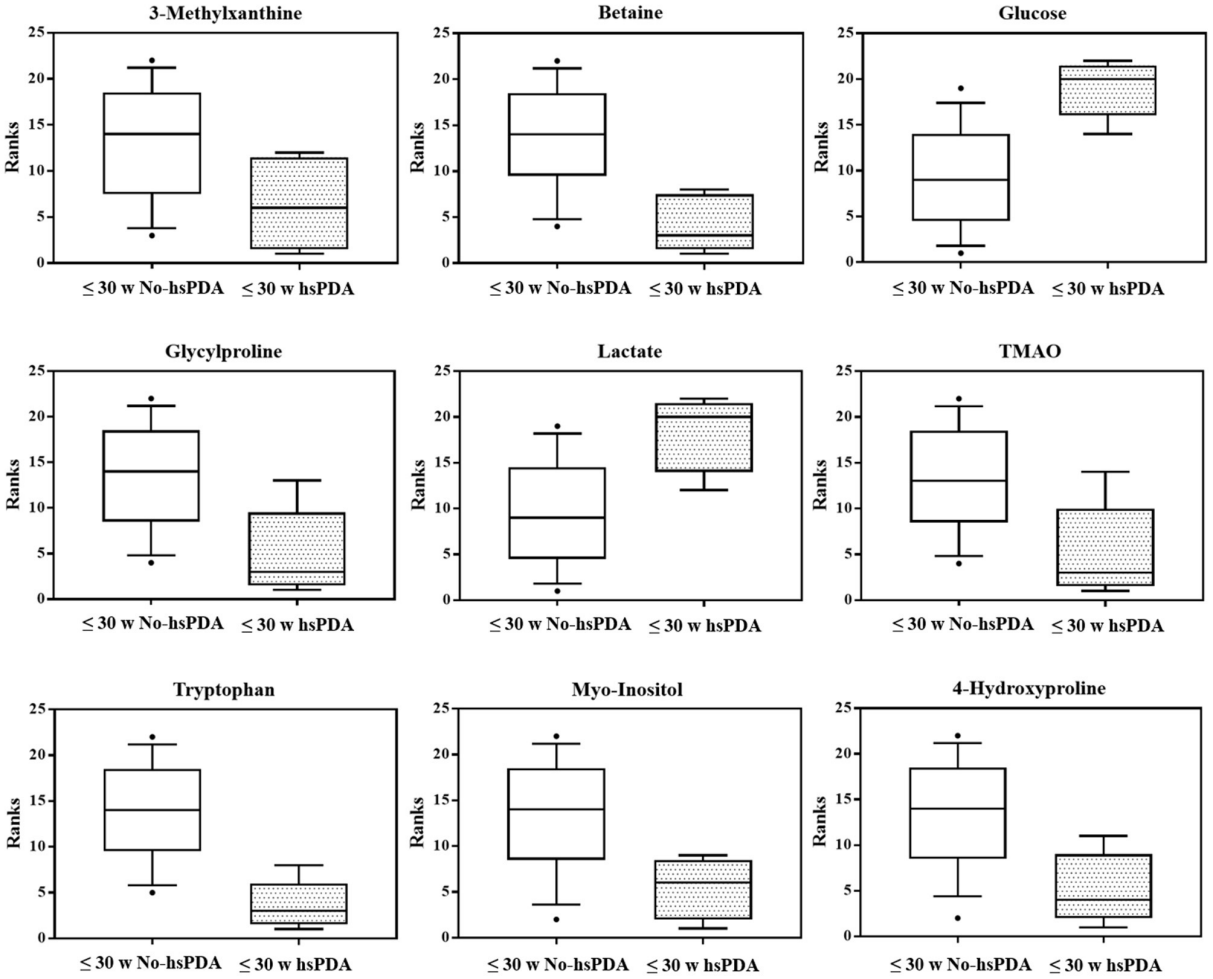
Box-and-whisker plots showing progressive changes of the metabolites concentration on ≤30 w No-hsPDA and ≤30 w hsPDA urine samples. Statistical significance was determined using the Mann-Whitney U-test (two-tailed) and a *p*-value < 0.05 was considered statistically significant. The Benjamini-Hochberg adjustment was applied.

**Figure 5:**
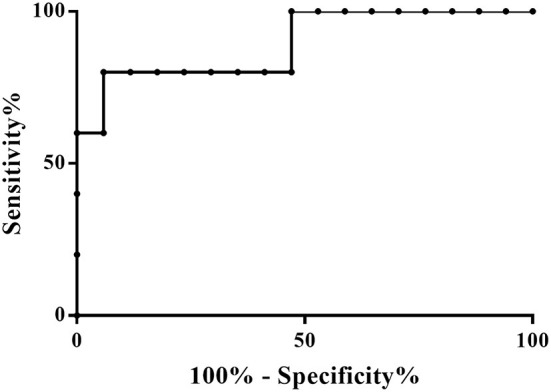
Representative ROC curves based on metabolites with *p*-value < 0.01 between ≤30 w No-hsPDA and ≤30 w hsPDA subjects. AUC: 0.894; Standard Error: 0.09; 95% CI: 0.717–1; *p*-value: 0.0087.

The PLS-DA scores plot (Figure 1) shows a marked separation along t(1) component between No-hsPDA (≤ and >30 w) and hsPDA (≤30 w) subjects, while along t(2) it is possible to observe a separation due to the different gestational age between No-hsPDA (≤30 w) and No-hsPDA (>30 w) subjects. The PLS-DA model was established with two components and showed good values of R2X, R2Y, and Q2 (Table 4). The validity of the PLS-DA model was evaluated through a permutation test (Supplementary Figure 1) using 400 times. The test results are reported in Table 4 and indicate the statistical validity of the PLS-DA model. To understand if the hsDPA can be a relevant variable on the urinary metabolomics profile of the studied subjects, an OPLS-DA model was constructed by considering the No-hsPDA (≤ and >30 w) subjects as a single class. OPLS-DA scores plot (Supplementary Figure 2) shows that the No-hsPDA (≤ and >30 w) subjects are clearly separate from hsPDA (≤30 w), indicating how the metabolomics profile between the two groups is strongly influenced by the hsPDA. The statistical parameters for the OPLS-DA model are shown in Table 4. The validity of the OPLS-DA model was evaluated through a permutation test (Supplementary Figure 3) using 400 times. The permutation test results indicate the statistical validity of the OPLS-DA model (Table 4). In order to identify potential metabolites closely involved in the separation of samples due to hsPDA, No-hsPDA (>30 w) subjects were excluded from the analysis. An OPLS-DA analysis was conducted by comparing subjects of the same gestational age with or without hsPDA. OPLS-DA scores plot in Figure 2 shows how subjects with No-hsPDA (≤30 w) are clearly separated from subjects hsPDA (≤30 w), indicating differences in the metabolomics profile between the two groups. The OPLS-DA model was established with one predictive and two orthogonal components and showed good values of R2X, R2Y, and Q2 (Table 4). The permutation test results are reported in Table 4 and shown in Supplementary Figure 4. The S-line plot shown in the Figure 3, was used to highlight the potential metabolites that contributed to the urine profile modification in No-hsPDA (≤30 w) subjects compared to hsPDA (≤30 w). A *p*(corr) > 0.5 was selected as a significance level. The spectral regions identified in the S-line plot characterized by the superimposition of NMR signals corresponding to the different metabolites present in urine, were quantified with Chenomx NMR Suite 7.1. and subjected to the Mann-Whitney U-test to identify significant changes in the of metabolites concentration in the two groups. The result of the univariate statistical analysis showed as 3-Methylxanthine, betaine, glucose, glycylproline, lactate, trimethylamine N-oxide (TMAO), tryptophan, myoinositol and 4-Hydroxyproline changed significantly in No-hsPDA (≤30 w) compared to hsPDA (≤30 w) (Table 5). The Figure 4 shows the relative concentrations of metabolites, where No-hsPDA (≤30 w) subjects were characterized by a higher level of 3-Methylxanthine, betaine, glycylproline, TMAO, tryptophan, myoinositol and 4-Hydroxyproline, and lower of levels glucose and lactate compared to hsPDA (≤30 w). Finally, A ROC curve was constructed by using the only metabolites with *p*-value < 0.01 (TMAO, tryptophan, and glucose) and the area under the curve of ROC analysis (Figure 5) was found to be 0.894 (95% CI: 0.717–1), denoting a high predictive accuracy of the model.

**Table 4:** Parameters for PLS-DA and OPLS-DA models.

	**PLS-DA and OPLS-DA models**			Permutation (400 times)^*^		
**Groups**	**Components^a^**	**R2Xcum^b^**	**R2Ycum^c^**	**Q2cum^d^**	**R2 intercept**	**Q2 intercept**
≤30 w No-hsPDA vs. >30 w No-hsPDA	2	0.669	0.819	0.501	0.474	−0.508
vs. ≤30 w hsPDA						
≤ and >30 w No-hsPDA vs. ≤30 w hsPDA	1P + 2O	0.539	0.862	0.479	0.586	−0.594
≤30 w No-hsPDA vs. ≤30 w hsPDA	1P + 2O	0.512	0.888	0.433	0.511	−0.496

a *The number of Predictive and Orthogonal components used to create the OPLS-DA statistical models*.

b, c *R2X and R2Y indicated the cumulative explained fraction of the variation of the X block and Y block for the extracted components*.

d *Q2 cum values indicated cumulative predicted fraction of the variation of the Y block for the extracted components*.

* *An Q2 intercept value <0.05 are indicative of a valid model*.

**Table 5:** Discriminant metabolites for OPLS-DA model.

**Metabolites**	**Mean (SD) of group (mM)^a^**		***p*-value^b^**
	**≤30 w No-hsPDA**	**≤30 w hsPDA**	
3-Methylxanthine	0.53 ± 0.34	0.23 ± 0.18	0.05
4-Hydroxyproline	6.39 ± 2.22	3.44 ± 1.72	0.03
Betaine	1.55 ± 0.73	0.69 ± 0.20	0.02
Creatine	1.19 ± 0.82	0.94 ± 0.78	0.31
Glucose	10.8 ± 7.24	37.5 ± 22.1	0.01
Glycylproline	8.55 ± 3.26	4.17 ± 1.97	0.02
Hippurate	0.52 ± 0.29	0.38 ± 0.17	0.44
Lactate	2.87 ± 1.03	6.10 ± 2.5	0.02
Methylhistidine	2.29 ± 1.95	1.18 ± 0.92	0.59
Myo-Inositol	34.6 ± 11.9	19.2 ± 6.03	0.04
Taurine	11.0 ± 4.44	5.78 ± 2.22	0.70
TMAO^*^	8.15 ± 2.39	4.68 ± 2.20	0.01
Tryptophan	0.76 ± 0.24	0.39 ± 0.17	<0.01

a *Relative concentrations were calculated by normalization of the molar concentration of each metabolite to the total molar concentration of all 13 metabolites for each sample*.

b *Statistical significance was determined using the Mann-Whitney U-test (two-tailed) and a p-value <0.05 was considered statistically significant. The Benjamini-Hochberg adjustment was applied*.

* *Trimethylamine-N-Oxide*.

## Discussion

### Clinical Discussion

Our group confirmed that prematurity influences the occurrence of hsPDA, which resulted as more frequent at a lower GA (*p* < 0.001). BW, although lower in the ≤30 w hsPDA group, did not reach a statistical significance (*p* = 0.069).

First of all, we must underline that our population, although relevant and significant for a metabolomics study, is small for epidemiological consideration and our clinical results should be confirmed on larger samples before deducing significant associations.

In our sample of neonates with GA ≤ 30w, the Apgar scores were significantly different in the two groups, with the lowest values in the neonates ≤30 w hsPDA both at 1′ (*p* < 0.01) and at 5′ (*p* < 0.05); therefore, the incidence of perinatal asphyxia was higher in this group (*p* < 0.05), especially severe asphyxia (*p* < 0.01). The association between lower values of the Apgar score and hsPDA have also been observed in the studies of Pourarian et al. and Lee et al. (22, 25), although the significance of the first study is limited by the inclusion of any size of PDA in the analysis.

Due to their worse condition at birth, those neonates subsequently developing hsPDA, also compatible with their aforementioned lower GA, more frequently required delivery room resuscitation (*p* < 0.05).

According to these data, neonates subsequently developing hsPDA showed the worst cardio-respiratory conditions at birth; however, it is not easy to asses with certainty if those neonates show the worst perinatal outcomes and subsequently hsPDA due to their lower GA or if the asphyxia itself could predispose to the development of hsPDA, likely through altered mechanisms of hypoxia-reperfusion, the potential involvement of systemic oxidant stress, vascular impairment and hemodynamic changes.

Surfactant administration was performed in a lower number of subjects in ≤30 w no-hsPDA group vs. ≤30 w hsPDA (*p* < 0.05). Such a result seems in agreement with some recent literature results (22, 25, 26), especially sustaining the association between late surfactant administration (after 2 h of life) and hsPDA, due to the rapid reduction in pulmonary pressure that favors blood flow through PDA (left-to-right shunt), lung hyperflow and ductal persistence (29). Further studies are needed to clarify whether surfactant administration could increase the risk of hsPDA or PDA could have more possibilities to evolve in hsPDA in those preterm neonates with a higher early respiratory impairment at birth, lung hypoxia and alveolar damage, evidenced by an increased need for surfactant and a more severe RDS.

Sepsis and early aminoglycosides administration were not more frequent in one of the two groups of our sample, although in literature neonatal sepsis has been identified as a predisposing factor for hsPDA through systemic inflammation and consequent potential ductal relaxation, both *via* respiratory failure and hypoxia, inflammatory mediators, and eventual aminoglycosides administration (27, 28).

Since aminoglycosides clearance can be reduced in premature neonates, in our NICU we measure the “peak” and “trough” serum levels after administration, to reduce the dose or prolong therapeutic intervals for the following administrations, performing a “tailored” therapeutic regimen (92).

Although renal function could be early impaired by hsPDA (93–95), in our sample, no statistically significant differences in creatinine values within the first 5 days of life occurred in the two groups.

However, serum creatinine increase can be delayed and only occur after a significant kidney injury, not representing a sensible and precocious marker (96).

### Metabolomics Discussion

According to our findings, metabolomics pathways characterizing urinary samples collected in the early period after birth seem highly dependent on neonatal probability to develop hsPDA confirmed at 48–72 h of life.

In detail, analyzing the whole number of urinary samples (*n* = 35) and as evidenced in Figure 1, there is a strong separation between the metabolomics profile of those newborns subsequently showing hsPDA (*n* = 5/35, red circles) and neonates no-hsPDA (*n* = 30, blue and green circles). Moreover, this urinary analysis also shows a clear dependence on GA, which increases in the newborns moving from the top to bottom of the plot: in fact, as showed in Figure 1, among the neonates no-hsPDA, the subjects represented in blue were born at GA ≤ 30w (*n* = 19/35), while those represented in green all show GA > 30 w.

From this analysis, it can be deduced that urinary samples at birth are strongly different in neonates subsequently developing hsPDA, all showing GA ≤ 30w (*n* = 5/35) and therefore well-separated in the plot from the whole sample of the neonates no-hsPDA (*n* = 30, including ≤30 w no-hsPDA, *n* = 19, and GA > 30 w, *n* = 11) and even located in the upper half area of the plot with neonates GA ≤ 30w. In fact, the lower half of the plot is almost exclusively populated by neonates with GA > 30 w and the lower half of the plot is occupied by neonates of GA ≤ 30w (*n* = 24/35 in total) with the aforementioned clear separation between ≤ 30 no-hsPDA (blue) and ≤30 w hsPDA.

The clear separation between urinary profiles of neonates hsPDA (*n* = 5/35, in red) and no-hsPDA (*n* = 30/35 if taking into account GA ≤ 30w and GA > 30 w together, black circles) can be also observed in the plot of Supplementary Figure 2.

However, although we initially selected our population study according to the range of GA 24–32 weeks and therefore included in the analysis *n* = 35 subjects, we observed that none of the newborns GA > 30 w developed hsPDA. HsPDA was diagnosed only in newborns GA ≤ 30w. Thus, observing a metabolomics dependence of our samples on GA, as reported and discussed for Figure 1, we decided to exclude those neonates GA > 30 w from further analysis, reducing our sample to those neonates GA ≤ 30w, to have a more homogeneous population in terms of GA and to improve the significance of our metabolomics comparison between neonates developing hsPDA (≤30 w hsPDA) and those not developing hsPDA (≤30 w no-hsPDA).

By the multivariate analysis of the urine spectra, as evidenced in Figure 2, a clear separation between urinary metabolomics profiles between ≤30 w no-hsPDA (black circles) vs. ≤30 w hsPDA (red circles), occurred and represents one of the most important results of our study.

Moreover, in the plot, *n* = 2/19 subjects of the ≤30 w no-hsPDA group, although localized in the appropriate area of the plot (no-hsPDA) seem mildly distant from the other samples. Interestingly, they showed some unique clinical features, represented by a congenital viral infection of the TORCH group in one subject and early pulmonary hypertension treated with NO immediately after birth in the second.

Additionally, we decided to quantify the most characterizing metabolites responsible for sample separation. As reported in the results, the ≤30 w hsPDA group was characterized by lower levels of 3-methylxanthine, betaine, glycylproline, TMAO, tryptophan, myoinositol and 4-hydroxyproline, and higher levels of glucose and lactate.

Firstly, we considered the potential interference of drugs in our group of neonates, by evaluating all the molecules and excipients administered (antibiotics, vitamins, parenteral nutrition, and other eventual drugs). Timing and modality of sampling were standardized and all the newborns underwent the same nutrition regimen.

Then, we tried to give a meaning to metabolomics variations, taking into account that there are not similar studies in literature.

#### Lactate

The significantly higher levels of urinary lactate observed in hsPDA group could be determined by the increase in anaerobic glycolysis following oxygen reduction and ischemia, with lactate being the end product of anaerobic metabolism (97, 98). We should understand if anaerobic metabolism determining lactate increase could be related to perinatal asphyxia and RDS, as reported by Ma et al. (99), or could be an early consequence of ductal steal and blood flow through PDA determining systemic hypoperfusion or depend on a not fully clarified interplay of these two mechanisms.

Lactate could also represent a compensatory and protective metabolite facing the increase in oxidative stress induced by reperfusion after ischemic damage, potentially lowering the levels of reactive oxygen species and superoxide anion (97, 98).

Lactate increase, following hypoperfusion and lactic acidosis by ductal steal, could also be one of the causes sustaining hsPDA, *via* the effects of acidosis and oxidant mechanisms linked to reperfusion on vasodilation and ductal relaxation (63).

Systemic hypoperfusion involves most organs including the brain; in this regard, the neuroprotective action of lactate, especially facing cerebral ischemia, was recently reported (100–106).

In some experiments involving animal models, hypoxic-ischemic encephalopathy was followed by an increased local level of lactate and glucose, sustaining their potential role as neuroprotectors (107, 108).

#### Glucose

In our hsPDA neonates, we observed a significant urinary increase in glucose levels. We hypothesized that glucose excretion, following its serum increase, could be determined by an increased stimulation of glucose-alanine cycle, since hypoxia-associated stress and an increased energy request could stimulate cortisol endogenous production (97). In hsPDA patients, a precocious ductal diastolic steal, even at birth, could be associated with an increase in oxygen consumption, also favored by RDS, influencing oxygen and glucose metabolism.

#### Myoinositol

It is synthesized from glucose-6-phosphate and represents a precursor of inositol (109). Intracellular myoinositol, a second messenger of insulin, was identified as a potential biomarker of impaired glucose metabolism (109–112).

Several factors regulate maternal, cord blood and neonatal myoinositol levels (110). Urinary levels of such metabolite at birth were identified as biomarkers of IUGR (112).

Since maternal myoinositol supplementation was shown to prevent neonatal hypoglycemia (110, 113), we hypothesized that urinary reduction of such metabolite in our hsPDA sample could be related to an increase in energy metabolism and glucose need.

Moreover, we suppose that its reduction could depend on alteration in glucose metabolism, possibly following a higher energy request.

During fetal life, myoinositol can increase to face oxidative stress in a low-oxigen environment (114, 115). Thus, we can also hypothesize that such metabolite is more consumed in hsPDA neonates, and therefore less excreted at urinary level, to improve antioxidant activity.

Finally, since myoinositol can also modify surfactant composition (110), our sampe of hsPDA neonates could have suffered of a more severe RDS at birth due to modifications in surfactant quality.

#### Betaine and TMAO

Betaine was significantly lower in hsPDA group. This metabolite, involved in several processes, derives from choline oxidation and acts as a methyl groups donor, also taking part in DNA methylation and signal transduction (97). It also serves as methyl donor for homocysteine during convertion into methionine; it can be assumed with diet or be synthesized through choline oxidation (109).

A potential link was observed between betaine ingestion and TMAO circulating levels, with pathways involving gut microbiota (116).

Among the intestinal microbes able to produce TMAO, we cite *A. hydrogenalis, C. asparagiforme, C. hateway* and others (117, 118).

Thus, the reduction in TMAO and betaine levels in our group of preterm hsPDA neonates, instead of no-hsPDA controls, seems to depend on modifications involving gut microbiota, due to the close link between these two metabolites and their interplay influencing intestinal microbes, in a not fully clarified way. This was also previously demonstrated in a metabolomics study on neonates developing BPD (97).

TMAO levels could be significantly influenced by antibiotics, due to their action on intestinal microflora (117).

However, in our group of neonates, no differences occurred in the administered antibiotics and dosages pro kilogram. Moreover, kidney function and creatinine levels did not show significant differences between the two groups, refuting the hypothesis of altered antibiotic metabolism.

TMAO and myoinositol urinary levels have been proposed to increase in case of renal dysfunction (98, 119–123).

Thus, we would have expected an increase in their levels in hsPDA neonates. On the contrary, they were significantly reduced, in disagreement with the hypothesis of a precocious kidney damage following ductal steal and renal hypoperfusion. In addition, the good creatinine levels observed in our hsPDA group, without significant differences instead of no-hsPDA group, would not sustain this hypothesis.

#### Tryptophan

It is an essential aminoacid taking part in several metabolic processes, such as nitrogen balance, growth, and production of niacin (a precursor of serotonin).

Tryptophan is mostly metabolized through the kynurenine pathway (90%); the 3% is converted into serotonin (5-OH-tryptamine) and the remaining quote undergoes intestinal microbiota degradation with production of indole and its derivatives (124).

Alterations in one of these processes could determine several effects in neuronal metabolic pathways.

Tryptophan and kynurenine could pass the blood-brain barrier and determine anti-inflammatory, immunosuppressive and antioxidant functions with neuroprotective actions (124).

We should also underline that tryptophan is reduced in neuroinflammatory diseases and neuroblastoma (124, 125), Alzheimer's and Parkinson's diseases (126, 127) and increased in neuropsychiatric disorders, such as autism (128).

In our sample, we speculated that reduced levels of such metabolite in the urines of hsPDA neonates could be related to cerebral hypoperfusion; the resulting oxidative/ischemic or inflammatory damage could determine an increase in tryptophan transport to the brain, with a reduced excretion of such metabolite, to improve neuroprotective effects. However, such compensatory mechanisms still need to be verified and eventually clarified.

Finally, the reduction observed in tryptophan level could also be related to microbial alterations, potentially modulating brain function.

#### 4-Hydroxyproline

It is a major constituent of collagen, contributing to its stabilization, and elastin. It is a non-essential aminoacid derived by hydroxylation of proline.

The increase in hydroxyproline levels (serum or urine) can be related to muscle damage (129) and collagen catabolism (bone reabsorption or tissue damage), even in response to oxidant stress. Such metabolite can be found altered in depression, Alzheimer disease and inborn errors of metabolism (130).

#### Glycylproline

This metabolite is a dipeptide composed by glycine and proline; it is involved in collagen metabolism. It can be mostly found in cytoplasm and it was correlated with Alzheimer's disease and inborn metabolic disorders too (131).

#### 3-Methylxanthine

It is one of the xanthine purine derivatives compounds; it can be mostly found in cytoplasm (132).

In humans, 3-methylxanthine, adenosine agonist, is a metabolite of theophylline (133) and caffeine (134), undergoes renal excretion without modifications (135).

In our sample, urinary 3-methylxantine can derive from the metabolism of caffeine administered to prevent prematurity-related apnea.

This metabolite, although not representing the most significant among our findings, was reduced in urinary samples of hsPDA neonates.

The newborns of the two groups (no-hsPDA and hsPDA) assumed the same dose of caffeine, thus the reason of such different excretions is not clear.

According to literature data, caffeine and theophylline metabolism are influenced by hepathic and renal function, in addition to some cytochrome polymorphisms (136).

The exact meaning of the decrease in 4-hydroxyproline, glycyproline and 3-methylxanthine in our sample of hsPDA neonates still requires clarification.

In conclusion, the early evaluation of neonatal urine to predict at birth the subsequent development of hsPDA should include the three “top metabolites” (glucose, thryptophan, and TMAO), which resulted the most significant in our preliminary results (*p* < 0.01). According to our study, we can affirm that the increase in glucose and the decrease in tryptophan and TMAO resulted the best predictors of hsPDA.

It would be interesting, even if not clinically appropriate, to perform brain magnetic resonance spectroscopy (MRS) to assess if biochemical changes in the brain agree with the urinary variations reported.

According to our results, metabolomics seems to have the unique ability to predict, in a sensible and specific way, those preterm newborns who will undergo physiological closure of DA or will maintain a no-hsPDA, instead of those neonates who will develop a hsPDA confirmed at 48–72 h of life, only through a non-invasive collection of a small urine sample at birth undergoing ^1^H-NMR evaluation.

We underline that our analysis was performed on the urine of the first 12 h of life and the metabolomics results can be considered a portrait of the metabolic basal status of each newborn. The role in predicting the subsequent development of hsPDA, if confirmed in future studies on a larger cohort of patients, could represent a unique peculiarity of metabolomics, able to individuate those newborns more predisposed to hsPDA before the occurrence of lung hyperflow clinical signs.

Such metabolomics approach could have the promising role to help in predicting those neonates who could develop hsPDA, preventing delay in therapeutic treatment, at the same time avoiding treatment to those who probably would not require it due to the spontaneous ductal closure.

This is the first time that metabolomics was applied in the early detection of hsPDA, and we decided to evaluate urinary samples due to the non-invasiveness of urine collection, being performed in the first hours after birth, and even taking into account the experience of our research group on urinary metabolomics in neonatology (97, 109, 137–140).

In our opinion, this kind of experimental study opens the way for future studies on a greater number of samples, possibly also multicenter studies, and the results, if confirmed, could lead to the individualization of clinical approaches in such field.

The analysis of seriate urinary samples before, during and after treatment, in subjects developing hsPDA, could help in detecting potential biomarkers predicting therapy response or drug toxicity, allowing physicians to choose in a personalized way the drug to administer, and potentially reducing side effects and improving treatment efficacy.

Thus, we strongly believe that, in the near future, such preliminary results could acquire a clinical and therapeutic meaning in improving hsPDA treatment. Such an innovative approach would result very differently from generalized therapeutic prophylaxis, that can determine side effects of complications even in those subjects who will have spontaneously achieved ductal closure.

The exact meaning of the metabolomics variation in our sample still requires a full comprehension since we only made some hypotheses and clinical speculation on the basis of what is currently known oh hsPDA pathophysiology.

In fact, this is the first available study on such a topic and our results, although promising, are preliminary reports on a limited number of samples that should be confirmed on larger groups.

However, despite these limitations, we believe that metabolomics is highly promising in the field of hsPDA, providing a combination of specific metabolites, also considering that, despite years of study on this topic, sensible and specific markers of such a condition are still lacking. In our opinion, the predictive power of metabolomics could result as potentially superior to the clinical or laboratory predictive tools explored to date.

## Conclusions

If our preliminary data will be confirmed, in the future, a simple and non-invasive urinary stick at birth could gave relevant and predictive information on the spontaneous closure or persistence of ductus arteriosus. This could be highly promising, given the great impact of such condition on immediate and long-term health, even taking into account the concept of perinatal programming. Metabolomics approach could help the clinicians to orient their therapeutic strategies in the management of such vulnerable newborns, early identifying those who could develop a hsPDA and therefore avoiding delaying treatment in such subjects, as well as, at the same time, identifying groups of newborns in which this condition is unlikely to occur and the therapy would therefore result in an overtreatment, not exempt from toxicity and side effects.

In our opinion, it could be useful to perform a subsequent study to confirm these data on larger samples and even to evaluate urinary profiles in relation to the kind, timing, and response to the treatment.

If these results will be confirmed, metabolomics could become a good tool in the early detection of hsPSA neonates, showing a stronger predictive power than clinical findings and laboratory parameters investigated to date, to optimize their management thanks to specific and holistic biomarkers in a field currently full of controversies.

## Data Availability Statement

The raw data supporting the conclusions of this article will be made available by the authors, without undue reservation.

## Ethics Statement

The studies involving human participants were reviewed and approved by Ethic Committee University of Cagliari. Written informed consent to participate in this study was provided by the participants' legal guardian/next of kin.

## Author Contributions

FB selected the patients, collected urinary samples, reviewed literature, and wrote the manuscript. CP performed metabolomics experiments, metabolomics data analysis, and wrote the manuscript. PN and AA contributed in writing the manuscript and performed echocardiograms. VF supervised the whole experiment and critically revised the manuscript. All authors contributed to the article and approved the submitted version.

## Conflict of Interest

The authors declare that the research was conducted in the absence of any commercial or financial relationships that could be construed as a potential conflict of interest.

## Supplementary Material

Supplementary Figure 1 Validation plots of OPLS-DA model (≤30 w No-hsPDA, >30 w No-hsPDA and ≤30 w hsPDA) using a permutation test. The horizontal axis shows the correlation between the permuted and actual data, while the vertical axis displays the cumulative values of R_2_ and Q_2_. The intercept gives an estimate of the overfitting phenomenon.

Supplementary Figure 2 OPLS-DA scores plot of ^1^H NMR spectra of urine samples: ≤30 w No-hsPDA and >30 w No-hsPDA (black circle), and ≤30 w hsPDA (red circle).

Supplementary Figure 3 Validation plots of OPLS-DA model (≤ and >30 w No-hsPDA and ≤30 w hsPDA) using a permutation test. The horizontal axis shows the correlation between the permuted and actual data, while the vertical axis displays the cumulative values of R_2_ and Q_2_. The intercept gives an estimate of the overfitting phenomenon.

Supplementary Figure 4 Validation plots of OPLS-DA model (≤30 w No-hsPDA and ≤30 w hsPDA) using a permutation test. The horizontal axis shows the correlation between the permuted and actual data, while the vertical axis displays the cumulative values of R_2_ and Q_2_. The intercept gives an estimate of the overfitting phenomenon.
