# Supplementary figures and images for: Early Urinary Metabolomics in Patent Ductus Arteriosus Anticipates the Fate: Preliminary Data
Source: Front Pediatr. 2020 Dec 21;8:613749. doi: 10.3389/fped.2020.613749 (PMC7779766; doi:10.3389/fped.2020.613749)

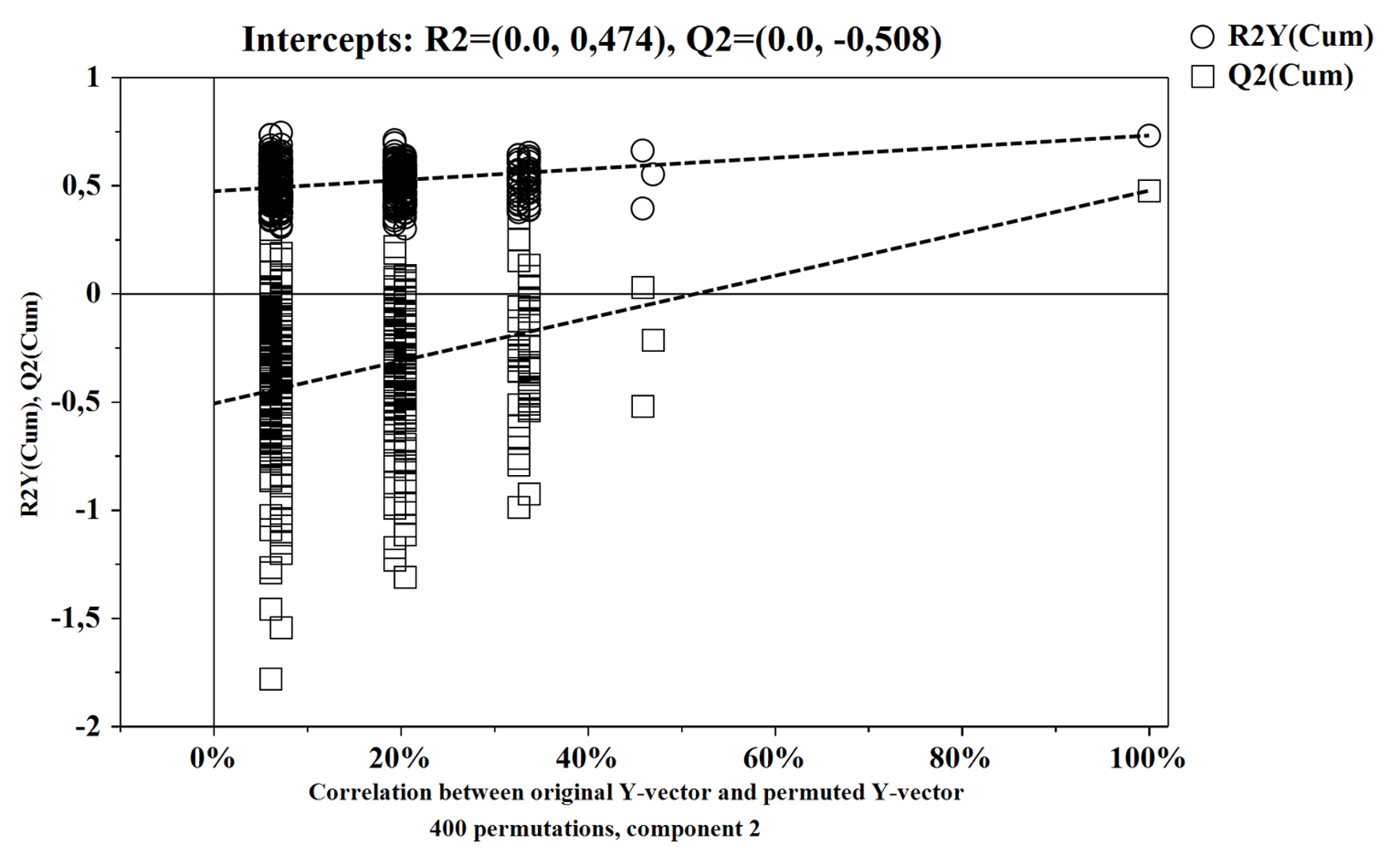

Supplement: Supplementary Figure 1 — Validation plots of OPLS-DA model (≤30 w No-hsPDA, >30 w No-hsPDA and ≤30 w hsPDA) using a permutation test. The horizontal axis shows the correlation between the permuted and actual data, while the vertical axis displays the cumulative values of R2 and Q2. The intercept gives an estimate of the overfitting phenomenon. [file Figure_1.TIF]

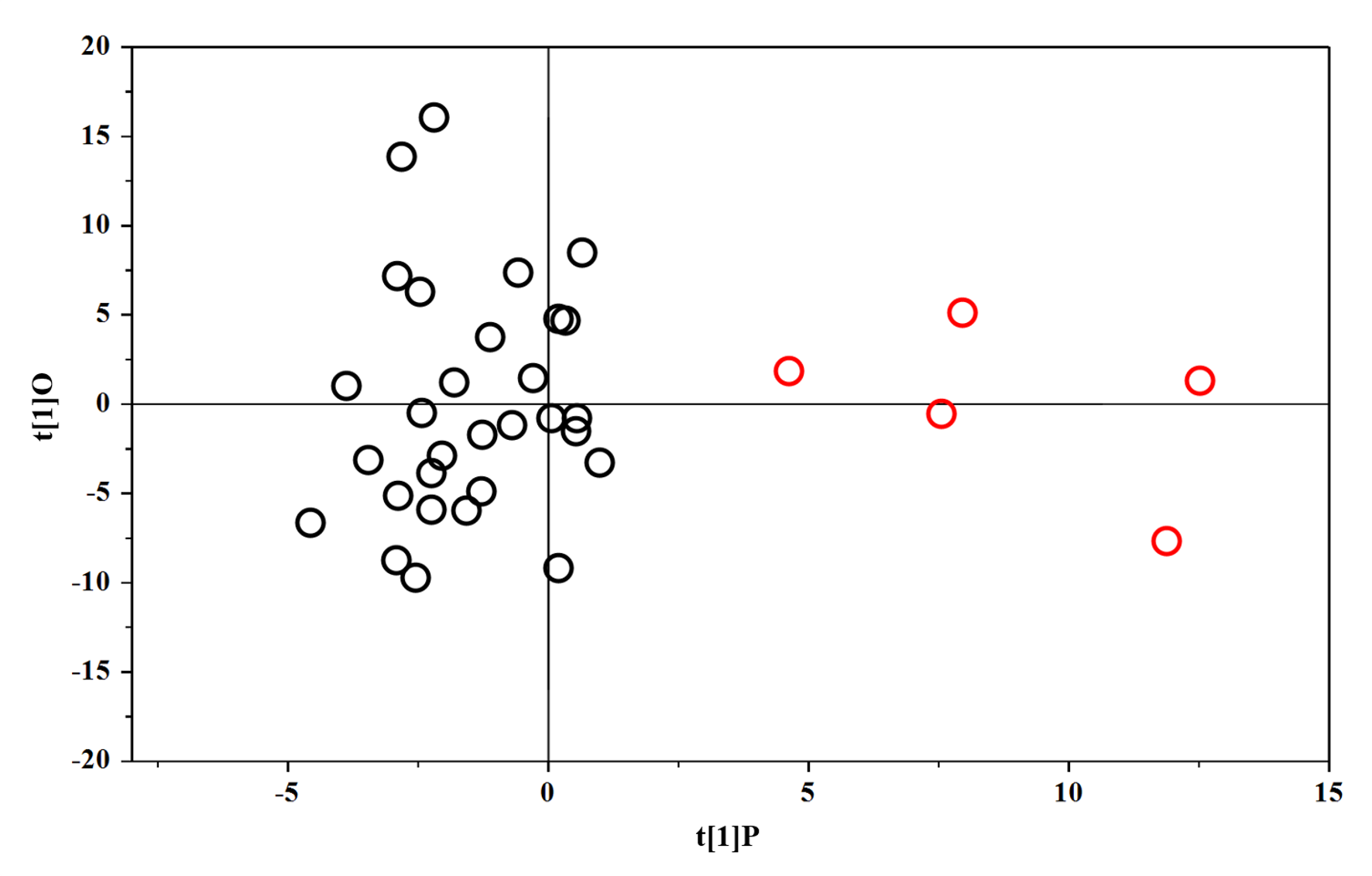

Supplement: Supplementary Figure 2 — OPLS-DA scores plot of 1H NMR spectra of urine samples: ≤30 w No-hsPDA and >30 w No-hsPDA (black circle), and ≤30 w hsPDA (red circle). [file Figure_2.TIF]

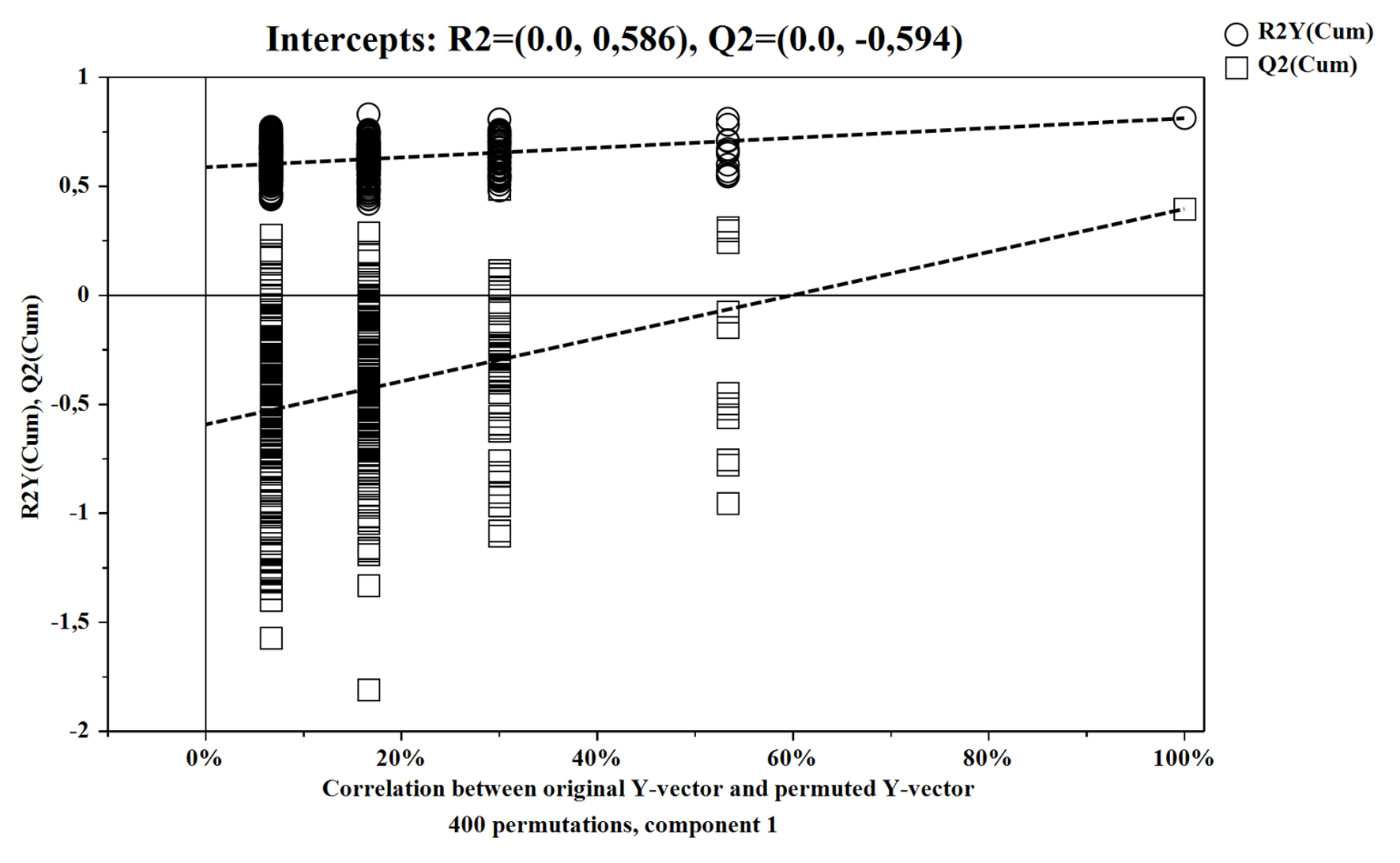

Supplement: Supplementary Figure 3 — Validation plots of OPLS-DA model (≤ and >30 w No-hsPDA and ≤30 w hsPDA) using a permutation test. The horizontal axis shows the correlation between the permuted and actual data, while the vertical axis displays the cumulative values of R2 and Q2. The intercept gives an estimate of the overfitting phenomenon. [file Figure_3.TIF]

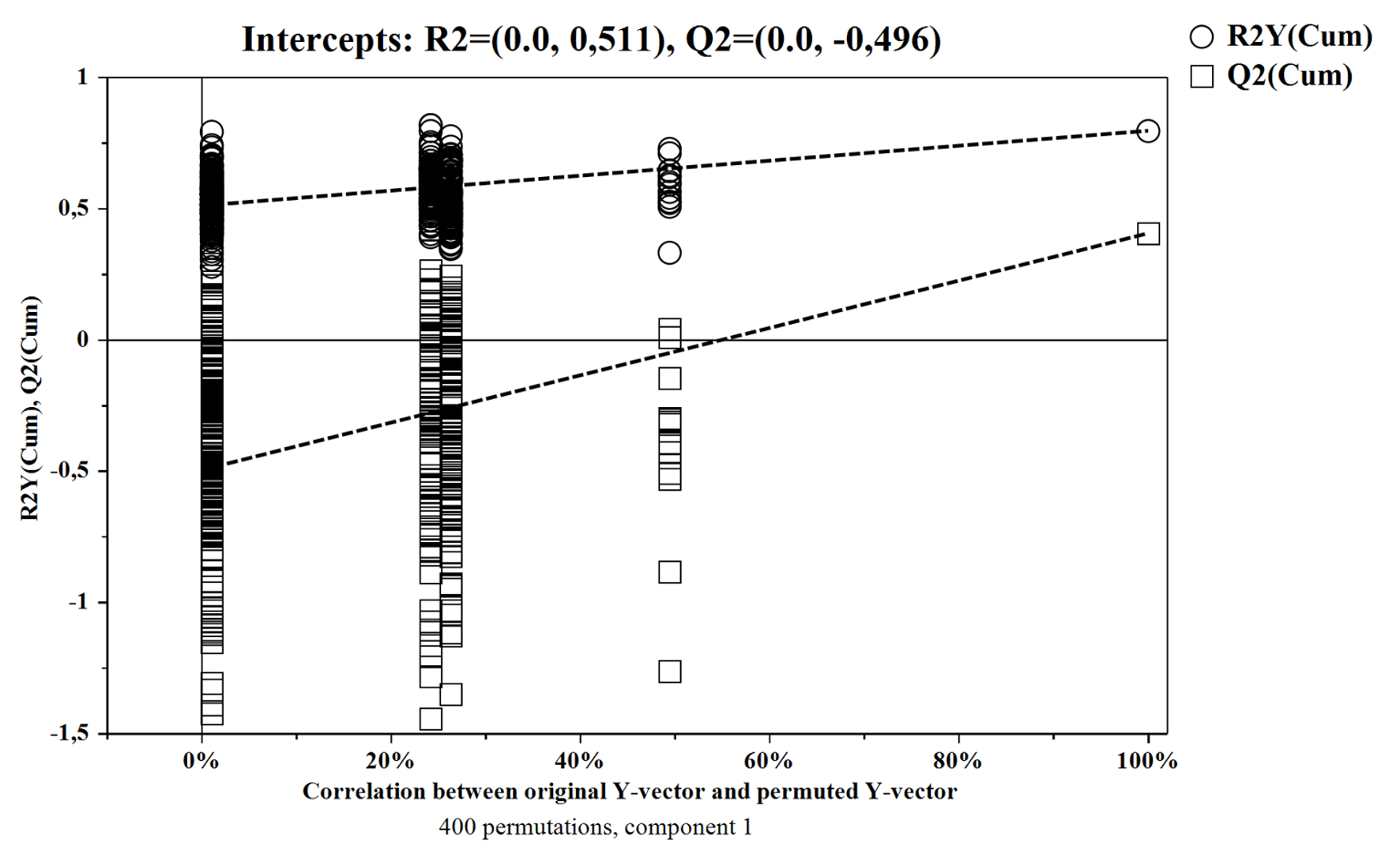

Supplement: Supplementary Figure 4 — Validation plots of OPLS-DA model (≤30 w No-hsPDA and ≤30 w hsPDA) using a permutation test. The horizontal axis shows the correlation between the permuted and actual data, while the vertical axis displays the cumulative values of R2 and Q2. The intercept gives an estimate of the overfitting phenomenon. [file Figure_4.TIF]
